# ZNF479 downregulates metallothionein-1 expression by regulating ASH2L and DNMT1 in hepatocellular carcinoma

**DOI:** 10.1038/s41419-019-1651-9

**Published:** 2019-05-28

**Authors:** Yi-Ju Wu, Bor-Sheng Ko, Shu-Man Liang, Yi-Jhu Lu, Yee-Jee Jan, Shih-Sheng Jiang, Song-Kun Shyue, Linyi Chen, Jun-Yang Liou

**Affiliations:** 1 Institute of Cellular and System Medicine, National Health Research Institutes, 350 Zhunan, Taiwan; 2 Institute of Molecular Medicine, National Tsing Hua University, 300 Hsinchu, Taiwan; 3 Department of Internal Medicine, National Taiwan University Hospital, 100 Taipei, Taiwan; 4 Department of Pathology and Laboratory Medicine, Taichung Veterans General Hospital, 407 Taichung, Taiwan; 5 National Institute of Cancer Research, National Health Research Institutes, 350 Zhunan, Taiwan; 6 Institute of Biomedical Sciences, Academia Sinica, 115 Taipei, Taiwan; 7 Graduate Institute of Biomedical Sciences, China Medical University, 404 Taichung, Taiwan

**Keywords:** Liver cancer, Cell growth

## Abstract

Decreased expression of metallothionein-1 (MT-1) is associated with a poor prognosis in hepatocellular carcinoma (HCC). Here, we found that MT-1 expression was suppressed by 14-3-3ε, and MT-1 overexpression abolished 14-3-3ε-induced cell proliferation and tumor growth. We identified that 14-3-3ε induced expression of ZNF479, a novel potential transcriptional regulator by gene expression profiling and ZNF479 contributed to 14-3-3ε-suppressed MT-1 expression. ZNF479 induced the expression of DNMT1, UHRF1, and mixed-lineage leukemia (MLL) complex proteins (ASH2L and Menin), and increased tri-methylated histone H3 (H3K4me3) levels, but suppressed H3K4 (H3K4me2) di-methylation. ZNF479-suppressed MT-1 expression was restored by silencing of ASH2L and DNMT1. Furthermore, ZNF479 expression was higher in HCC tissues than that in the non-cancerous tissues. Expression analyses revealed a positive correlation between the expression of ZNF479 and DNMT1, UHRF1, ASH2L, and Menin, and an inverse correlation with that of ZNF479, ASH2L, Menin, and MT-1 isoforms. Moreover, correlations between the expression of ZNF479 and its downstream factors were more pronounced in HCC patients with hepatitis B. Here, we found that ZNF479 regulates MT-1 expression by modulating ASH2L in HCC. Approaches that target ZNF479/MLL complex/MT-1 or related epigenetic regulatory factors are potential therapeutic strategies for HCC.

## Introduction

Metallothioneins (MTs) are a group of low molecular weight (~6 kDa) polypeptides that contain cysteine-rich sequences with a high affinity for heavy metals and oxidative stress^1,2^. The expression levels of MTs are induced by heavy metals, cytokines, and ROS. The MT-1 proteins function as scavengers to directly intercept heavy metals and reactive oxygen species that may damage cells or tissues^2^. MT-1 expression is increased in breast cancer, non-small-cell lung cancer, ovarian cancer, pancreatic carcinoma, renal carcinoma, and skin carcinoma, but decreased in colorectal carcinoma, gastric carcinoma, prostate cancer, and small-cell lung cancer^3^. MT-1 proteins are abundantly expressed in the liver, but are downregulated in hepatocellular carcinoma (HCC)^4,5^. Methylation of the *MT-1M* and *MT-1G* promoters is significantly increased in HCC patients, and this is positively correlated with tumor size and the incidence of metastases^6^. Moreover, overexpression of MT-1M could reduce cell proliferation and tumor growth in HCC^7^. Therefore, reduced expression of MT-1 is a potential diagnostic marker for HCC.

The stringent reduction of MT1 expression level in HCC raises the possibility that its promoters and other important *cis*-elements are epigenetically modified to achieve long-term repression, which can be regulated via specific histone modifications and DNA hypermethylation (Table. 1). The mixed-lineage leukemia (MLL) complex displays histone methyltransferase activity and helps to regulate transcription^8^. Absent, small, or homeotic discs-like protein (ASH2L), a component of the MLL complex, functions as an oncoprotein^9^ and overexpression of ASH2L stimulates the methyltransferase activity of the MLL complex^10^. ASH2L is essential for tri-methylation of H3K4 (H3K4me3) as lacking of ASH2L in the MLL complex reduces H3K4me3 levels but increases H3K4 di-methylation (H3K4me2)^11^. In addition, MLL complex is recruited to the chromatin via interacts with a histone-binding factor called Menin^8,12^. Expression of Menin is significantly associated with liver fibrogenesis and is correlated with that of ASH2L in HCC^13,14^. Moreover, increased Menin expression has been found to promote hepatocellular carcinogenesis^13^. Therefore, it will be interesting to know how MLL complex and associated factors are involved in the carcinogenesis of HCC.

**Table 1:** Correlation between ZNF479 expression and clinicopathologic characteristics of HCC patients

Characters	*N*	LogT_mRNA_–logN_mRNA_	*p*-value
Total	150	0.27 ± 0.83	
Age			
≤60 y/o	68	0.31 ± 0.87	NS
>60 y/o	82	0.24 ± 0.80	
Gender			
Male	102	0.32 ± 0.87	NS
Female	48	0.16 ± 0.74	
Smoking			
Yes	72	0.28 ± 0.85	NS
No	75	0.29 ± 0.81	
Unknown	3		
Drinking			
Yes	31	0.61 ± 0.85	0.013
No	116	0.20 ± 0.80	
Unknown	3		
Tumor size (diameter)			
≤5cm	70	0.43 ± 0.81	0.032
>5cm	80	0.14 ± 0.83	
Pathology type			
Solitary	103	0.29 ± 0.82	NS
Multiple	46	0.26 ± 0.84	
Infiltrative	1	−1.24	
Vascular invasion			
Absent	49	0.63 ± 0.80	0.001
Capsular vein invasion	18	0.25 ± 0.84	
Portal vein invasion	83	0.07 ± 0.78	
AJCC staging			
Stage I	41	0.63 ± 0.79	0.007
Stage II	57	0.21 ± 0.79	
Stage III	47	0.08 ± 0.87	
Stage IV	5	−0.11 ± 0.45	
BCLC staging			
Stage A	59	0.54 ± 0.80	0.002
Stage B	58	0.20 ± 0.86	
Stage C	33	−0.07 ± 0.83	
Cirrhosis			
Yes	46	0.55 ± 0.94	0.006
No	104	0.75 ± 0.74	
Alpha-fetoprotein			
≤80 ng/ml	92	0.30 ± 0.78	NS
>80 ng/ml	58	0.22 ± 0.91	
Viral infection			
Hepatitis B	50	0.46 ± 0.98	NS
Hepatitis C	50	0.14 ± 0.68	
No hepatitis B or C	50	0.22 ± 0.79	
Metastasis			
Yes	9	−0.05 ± 0.39	NS
No	141	0.29 ± 0.85	

DNA methyltransferases (DNMTs) contribute to gene regulation in mammalian cells by epigenetic modification. DNMT1 and DNMT3a are involved in the early stages of hepatocarcinogenesis^15^, and increased expression of DNMT1 is significantly correlated with malignancy and poor prognosis of human HCC^16^. It has been shown that treatment with 5-azacytidine (5-AzaC) decreased the association of DNMT1 with the *MT-1* promoter, thereby inducing MT-1 expression and restoring *MT-1* promoter activity in hepatoma cells^17,18^. Additionally, DNMT1 is a target of microRNA-140 (miR-140), and it has been reported that miR-140^–/–^ mice show increased DNMT1 expression accompanied by reduced MT-1 expression^19^. These results suggest that DNMT1 plays an important role on regulating DNA methylation and *MT1* promoter activity. In addition, DNMT1 binds to the CpG islands of the *cis*-elements through interacting with ubiquitin-like with PHD and ring finger domains 1 (UHRF1, also known as NP95 in mice and ICBP90 in humans), which maintains DNA methylation by associating with methylated histone H3^20–23^. The expression of UHRF1 is increased in HCC and is associated with a poor prognosis as it promotes cell proliferation and metastasis^24^. However, whether UHRF1 expression associates with MT-1 expression has never been elucidated.

The Krüppel-associated box (KRAB) domain containing zinc finger proteins (KRAB-ZFPs) are the largest family of transcription repressors in mammals^25–27^. Although the functions of most KRAB-ZFPs are unknown, some have been shown to repress transposable elements by interacting with co-repressor tripartite motif-containing 28 (TRIM28; also known as KRAB-associated protein-1 [KAP-1])^28^. According to sequence homology, zinc finger protein 479 (ZNF479, also known as HKr19) is a potent KRAB-ZFP^29^. ZNF479 was first cloned from a human testis cDNA library^29^. However, the functions or regulation of ZNF479 has never been investigated.

14-3-3 proteins are implicated in regulating multiple cellular and physiological functions^30^. 14-3-3 proteins are overexpressed in HCC and regulate tumor progression^31–36^. In this work, we reveal that 14-3-3ε suppressed the expression of MT-1 via inducing ZNF479 expression. siRNA silencing of ZNF479 significantly induced MT-1 expression and attenuated 14-3-3ε-induced cell proliferation and tumor growth. Moreover, the suppression of MT-1 by ZNF479 was mediated by the induction of ASH2L and DNMT1. Results from expression analyses in clinical HCC tissues revealed that expression of ZNF479 is positively correlated with that of DNMT1, UHRF1, ASH2L, and Menin but inversely correlated with MT-1. Finally, we found that correlations between the expression of ZNF479 and its downstream factors are more pronounced in hepatitis B virus (HBV) positive patients. Thus, ZNF479 may play an important role in suppressing MT-1 expression and modulating HCC tumor progression by regulating components of the MLL complex and epigenetic modification.

## Results

### 14-3-3-suppressed MT-1 expression contributes to HCC cell proliferation and tumor growth

**Fig. 1:**
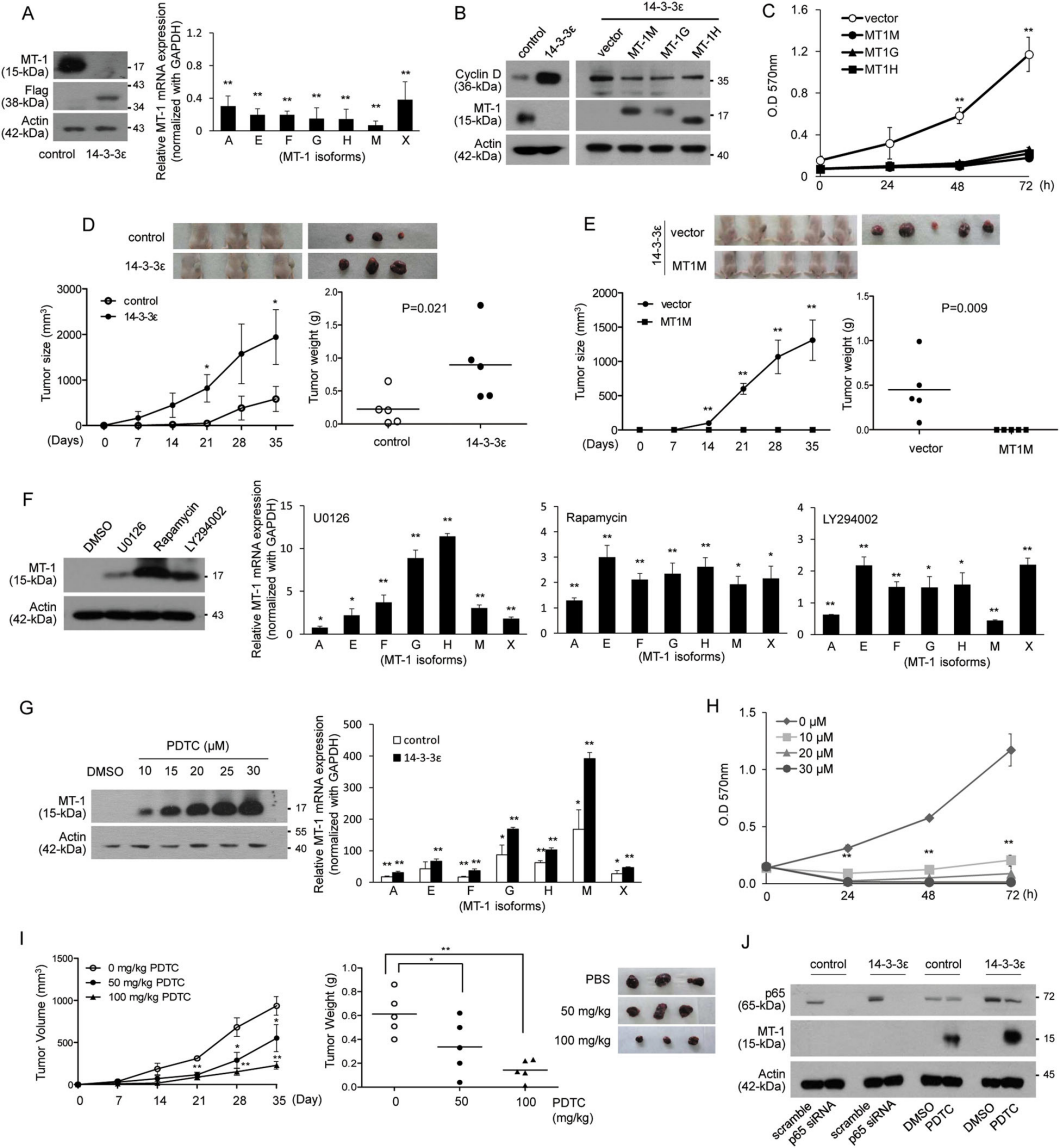
14-3-3ε–suppressed MT-1 expression contributes to cell proliferation and tumor growth. **a** Relative expression of MT-1 isoforms in Huh-7 cells was determined by western blot and qPCR analysis in 14-3-3ε–overexpressing stable cells (14-3-3ε). **b**, **c** 14-3-3ε cells were transiently transfected with MT-1 overexpression vectors. Expression of cyclin D and MT-1 was determined by western blot analysis and cell proliferation by MTT assay. **d** Tumor growth and weight in an in vivo xenograft mouse model of 14-3-3ε overexpression versus control cells, and **e** MT-1M overexpression versus control vector in 14-3-3ε cells. Tumor growth was examined by calculating tumor volume every week and tumor weight at day 35, *N* = 5 in each group. **f** 14-3-3ε–overexpressing cells were treated with 10 μg/μl U0126, 10 μM rapamycin, 20 μM LY294002, or DMSO for 24 h. Relative expression of MT-1 was determined by western blot and qPCR analysis. **g** 14-3-3ε–overexpressing cells were treated with 10–30 μM PDTC for 24 h. Expression of MT-1 was determined by western blot and qPCR analysis (30 μM of PDTC). **h** 14-3-3ε–overexpressing cells were treated with various concentrations of PDTC for 24–72 h. Cell proliferation was examined by MTT assay. **i** Nude mice were subcutaneously injected with 2 × 10^6^ 14-3-3ε–overexpressing cells for 1 week, followed by peri-tumoral injection with vehicle control (PBS) or PDTC (50 and 100 mg/kg) every 2 days for 4 weeks. Tumor growth was examined by tumor volume and tumor weight, *N* *=* 5 in each group. **j** Control and 14-3-3ε–overexpressing cells were transiently transfected with scramble or p65 siRNA for 24 h, then treated with 30 μM PDTC for 24 h. Expression of p65 and MT-1 was examined by western blot analysis. Gene expression of qPCR analysis was normalized to both control cells as well as *GAPDH*. Actin was used as the loading control for western blotting analysis. Scale bars: mean ± SD. *P < 0.05; **P < 0.01

The expression of MT-1 was determined by western blot analysis and validated by qPCR with MT-1 isoforms (Fig. S1) in Huh-7 and HepG2 cells transiently overexpressed with FLAG-tagged 14-3-3ε. The expression of MT-1 isoforms was reduced, and this was further confirmed in 14-3-3ε-overexpressing stable cells (Fig. 1a). Unexpectedly, siRNA silencing of 14-3-3ε exhibited no significant effect on MT-1 expression (Fig. S2). As 14-3-3ε may form heterodimers with other 14-3-3 isoforms, we suppressed 14-3-3 activity by delivery of a peptide-based 14-3-3 inhibitor, dimeric fourteen-three-three peptide inhibitor (difopein, peptide coding sequences:PHCVPRDLSWLDLEANMCLP), into 14-3-3ε-overexpressing cells. Overexpression of difopein significantly induced MT-1 expression (Fig. S3). To further validate that MT-1 isoforms are downstream factors of 14-3-3ε, we transiently transfected MT-1G, -1H, and -1M overexpression vectors into 14-3-3ε-overexpressing cells. 14-3-3ε overexpression induced cyclin D and reduced MT-1 expression (Fig. 1b). Overexpression of MT-1 attenuated 14-3-3ε-induced cyclin D expression (Fig. 1b, right panel), cell proliferation (Fig. 1c), and anchorage-independent cell growth (Fig. S4). In addition, we examined the effect of MT-1 overexpression on suppressing 14-3-3ε-induced HCC tumor growth in an in vivo xenograft mouse model. 14-3-3ε overexpression significantly induced tumor growth in nude mice (Fig. 1d), and this effect was abrogated by MT-1M overexpression (Fig. 1e).

### MEK/ERK, PI3K/Akt, and mTOR signaling involved in 14-3-3ε–suppressed MT-1 expression

14-3-3 proteins control various cellular functions through regulating distinct signal pathways^37^. We examined the potential signaling involved in 14-3-3ε-suppression of MT-1 expression using pharmacological inhibitors. SB216763 (inhibitor of glycogen synthase kinase-3), Y27632 (inhibitor of Rho-A kinase), and SP600125 (inhibitor of JNK) exhibited either no effect or only slightly influenced the expression of MT-1 isoforms (Fig. S5). However, U0126 (inhibitor of MEK) and rapamycin (inhibitor of mTOR) significantly induced the expression of most MT-1 isoforms in 14-3-3ε stable cells (Fig. 1f, left panel). LY294002 (inhibitor of PI3K) also induced the expression of most MT-1 isoforms, but partially reduced MT-1A and MT-1M expression (Fig. 1f, right panel). Additionally, U0126, rapamycin, and LY294002 dose-dependently reduced colony formation (Fig. S7). We had further examined the expression of phosphorylated signal kinases including ERK1/2 and Akt, which are commonly activated by 14-3-3 proteins. Expression of phosphorylated ERK1/2 was elevated in 14-3-3ε overexpressing cells (Fig. S6a). Moreover, as it has been demonstrated that Wnt signal is activated in HCC and β-catenin was regulated by 14-3-3ε^36^, we had confirmed that expression of β-catenin was increased in 14-3-3ε stable cells (Fig. S6a). However, treatment with DKK-1 (inhibitor of Wnt signaling) had no significant effect on MT-1 expression in 14-3-3ε stable cells (Fig. S6b and S6c).

### PDTC induces MT-1 expression, suppresses cell proliferation and tumor growth

While investigating the signaling pathways involved in regulating MT-1 expression, we found that PDTC dose-dependently and abundantly induced MT-1 expression (Fig. 1g), and significantly suppressed cell proliferation (Fig. 1h). We also found that peri-tumoral injection of PDTC into an in vivo xenograft mouse model significantly reduced tumor volume and weight (Fig. 1i). Because PDTC is commonly used as an NFκB inhibitor, we investigated whether NFκB is involved in regulating MT-1 expression. We found higher p65 expression in 14-3-3ε-overexpressing cells than in control cells (Fig. 1j). However, siRNA knockdown of p65 exhibited no effect on MT-1 expression (Fig. 1j).

### ZNF479 suppresses MT-1 expression and promotes cell proliferation

**Fig. 2:**
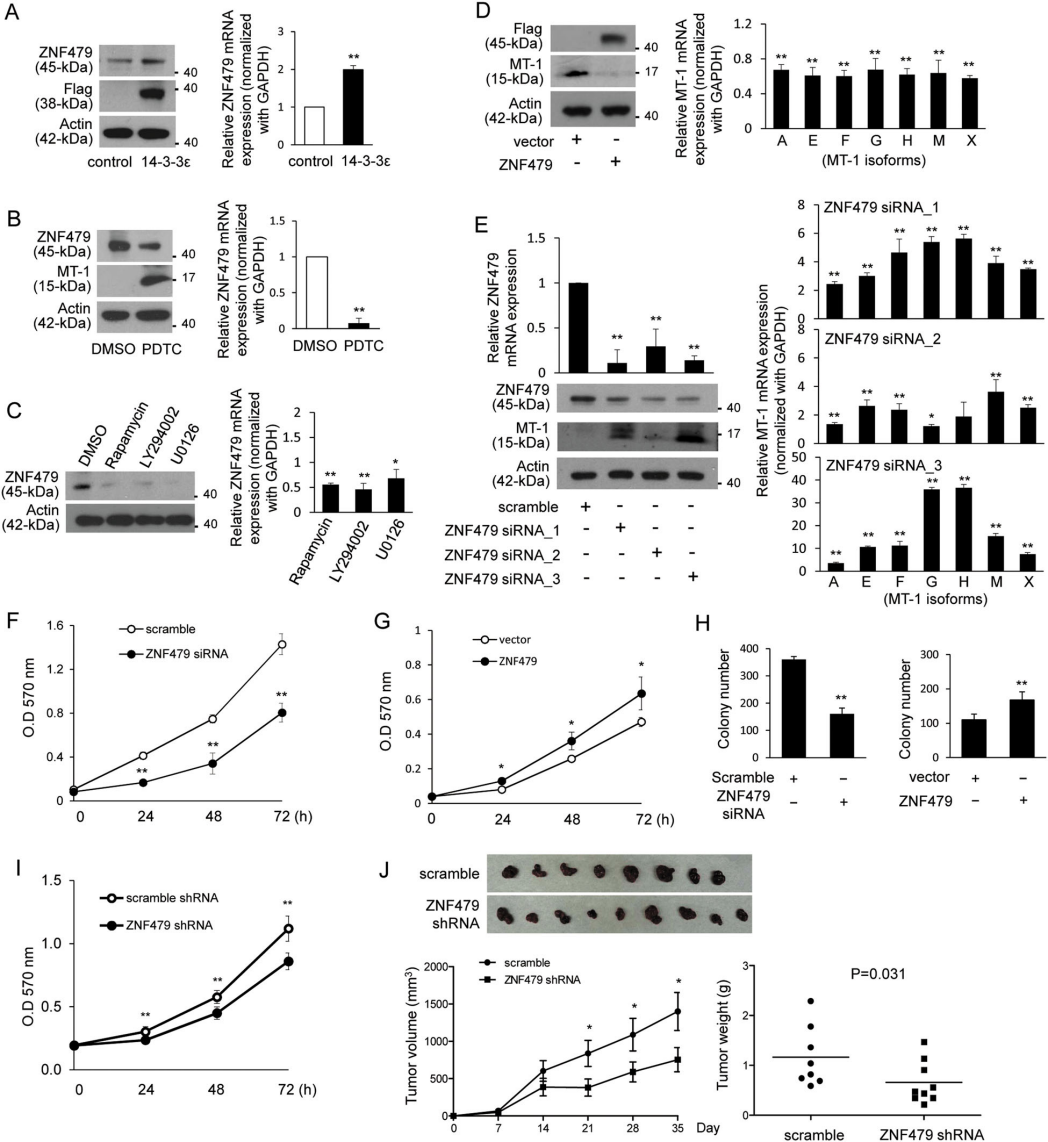
ZNF479 suppresses MT-1 expression and promotes cell proliferation and tumor growth. **a** Expression of ZNF479 in control and 14-3-3ε–overexpressing cells determined by western blot and qPCR analysis. **b** 14-3-3ε–overexpressing cells were treated with 30 μM PDTC, **c** 10 μM rapamycin, 20 μM LY294002, 10 μg/μl U0126, or DMSO for 24 h. Relative expression of ZNF479 and MT-1 was examined by western blot and qPCR analysis. **d** Huh-7 cells were transfected with a ZNF479 (FLAG-tagged) overexpression vector for 48 h. Expression of MT-1 was determined by western blot analysis. **e** 14-3-3ε–overexpressing cells were transfected with or without ZNF479 siRNA. Expression of ZNF479 and MT-1 was determined by western blot and qPCR analysis. **f** 14-3-3ε–overexpressing cells were transfected with ZNF479 siRNA. Cell proliferation was examined by MTT assay. **g** Huh-7 cells were transfected with a ZNF479 overexpression vector. Cell proliferation was examined by MTT and **h** colony formation assays. **i** Cell proliferation was examined by MTT assay in stable cells with ZNF479 shRNA treatment. **j** Nude mice were subcutaneously injected with 2 × 10^6^ cells with ZNF479 shRNA (*N* = 9) and scramble control (*N* = 8). Tumor growth was examined by tumor volume and tumor weight. Gene expression of qPCR analysis was normalized to both control cells as well as *GAPDH*. Actin was used as the loading control for western blotting analysis. Scale bars: mean ± SD. *P < 0.05; **P < 0.01

To investigate the potential transcriptional regulator modulating MT-1 expression, we performed gene expression profiling by microarray analysis in PDTC-treated 14-3-3ε-overexpressing cells (Table S1). We validated the mRNA expression of several transcriptional regulators by qPCR analysis and found that expression of early growth response protein 1 (EGR-1) but not zinc finger protein 578 (ZNF578) was induced in 14-3-3ε-overexpressing cells (Fig. S8a and S8b). PDTC treatment significantly suppressed EGR-1 expression (Fig. S8c and S8d). Although siRNA knockdown of EGR-1 induced MT-1 expression by qPCR analysis (Fig. S8e, left panels), EGR-1 silencing had no significant effect on the induction of MT-1 expression by western blotting analysis (Fig. S8e, right panel). Additionally, the expression of all MT-1 isoforms was abundantly induced by PDTC, but that of a zinc finger protein, ZNF479, was suppressed by PDTC treatment (Table S1). While 14-3-3ε induced ZNF479 expression (Fig. 2a), PDTC suppressed its expression (Fig. 2b). Moreover, rapamycin, LY294002, and U0126 significantly attenuated ZNF479 expression (Fig. 2c). Overexpression of ZNF479 in Huh-7 cells reduced MT-1 expression (Fig. 2d), and ZNF479 siRNA knockdown induced MT-1 expression (Fig. 2e).

To investigate whether ZNF479 contributes to the regulation of cell proliferation, we used ZNF479 siRNA in 14-3-3ε- and ZNF479-overexpressing Huh-7 cells. ZNF479 siRNA suppressed cell proliferation (Fig. 2f), and ZNF479 overexpression induced cell proliferation (Fig. 2g) and anchorage-independent cell growth (Fig. 2h). We confirmed our results with stable ZNF479 shRNA knockdown cells (Fig. S9), which showed reduced cell proliferation (Fig. 2i). Additionally, ZNF479 shRNA significantly reduced tumor size and weight in the in vivo mouse model (Fig. 2j).

### H3K4 methylation is involved in ZNF479-suppressed MT-1 expression

**Fig. 3:**
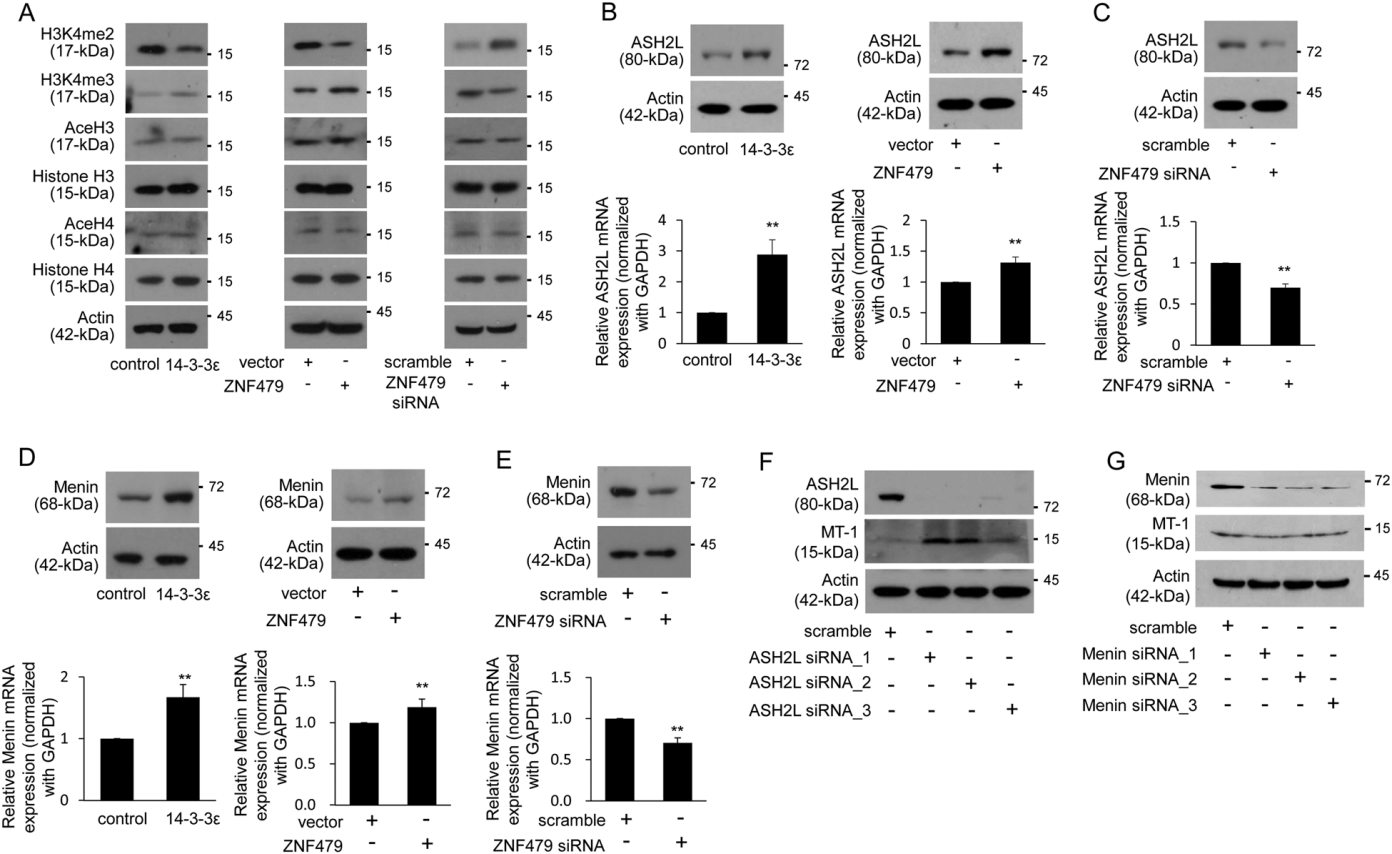
ASH2L is involved in ZNF479-suppressed MT-1 expression. Huh-7 cells were transfected with control/ZNF479 overexpression vectors or scramble/ZNF479 siRNAs for 48 h. **a** Expression of H3K4me2, H3K4me3, Acetyl-H3, Histone H3, Acetyl-H4, and Histone H4 was examined by western blot analysis in control/14-3-3ε–overexpressing cells (left), vector/ZNF479-transfected cells (middle) and scramble/ZNF479 siRNA-transfected cells (right). **b**–**e** Expression of ASH2L and Menin was examined by western blot and qPCR analysis in control/14-3-3ε–overexpressing cells (**b**, **d**, left), vector/ZNF479-transfected cells (right) and scramble/ZNF479 siRNA-transfected cells (**c**, **e**). **f**, **g** Huh-7 cells were transfected with ASH2L and Menin siRNAs for 48 h. Relative expression of ASH2L, Menin and MT-1 was determined by western blot analysis. Gene expression of qPCR analysis was normalized to both control cells as well as *GAPDH*. Actin was used as a loading control for western blotting analysis. Scale bars: mean ± SD. **P < 0.01

Because ZNF479 contains a KRAB domain that may contribute to epigenetic regulation^29^, we investigated whether histone methylation is involved in ZNF479-suppressed MT-1 expression. H3K4me2 levels were reduced and H3K4me3 levels were increased in the 14-3-3ε- and ZNF479-overexpressing cells (Fig. 3a, left and middle panels). ZNF479 siRNA knockdown demonstrated the opposite effect (Fig. 3a, right panel). As ASH2L, a crucial component of the MLL complex, is essential for regulating the transition between di- and tri-methylation of H3K4^11^, we examined whether the expression of ASH2L is regulated by ZNF479. Overexpression of 14-3-3ε and ZNF479 induced ASH2L expression (Fig. 3b). In contrast, ZNF479 siRNA attenuated ASH2L expression (Fig. 3c). 14-3-3ε and ZNF479 overexpression induced expression of the MLL-complex protein, Menin (Fig. 3d), and ZNF479 siRNA reduced Menin expression (Fig. 3e). Moreover, siRNA silencing of ASH2L expression induced MT-1 expression (Fig. 3f). However, silencing of Menin by siRNA had no significant effect on induction of MT-1 expression (Fig. 3g).

### DNMT1 contributes to ZNF479-suppressed MT-1 expression

**Fig. 4:**
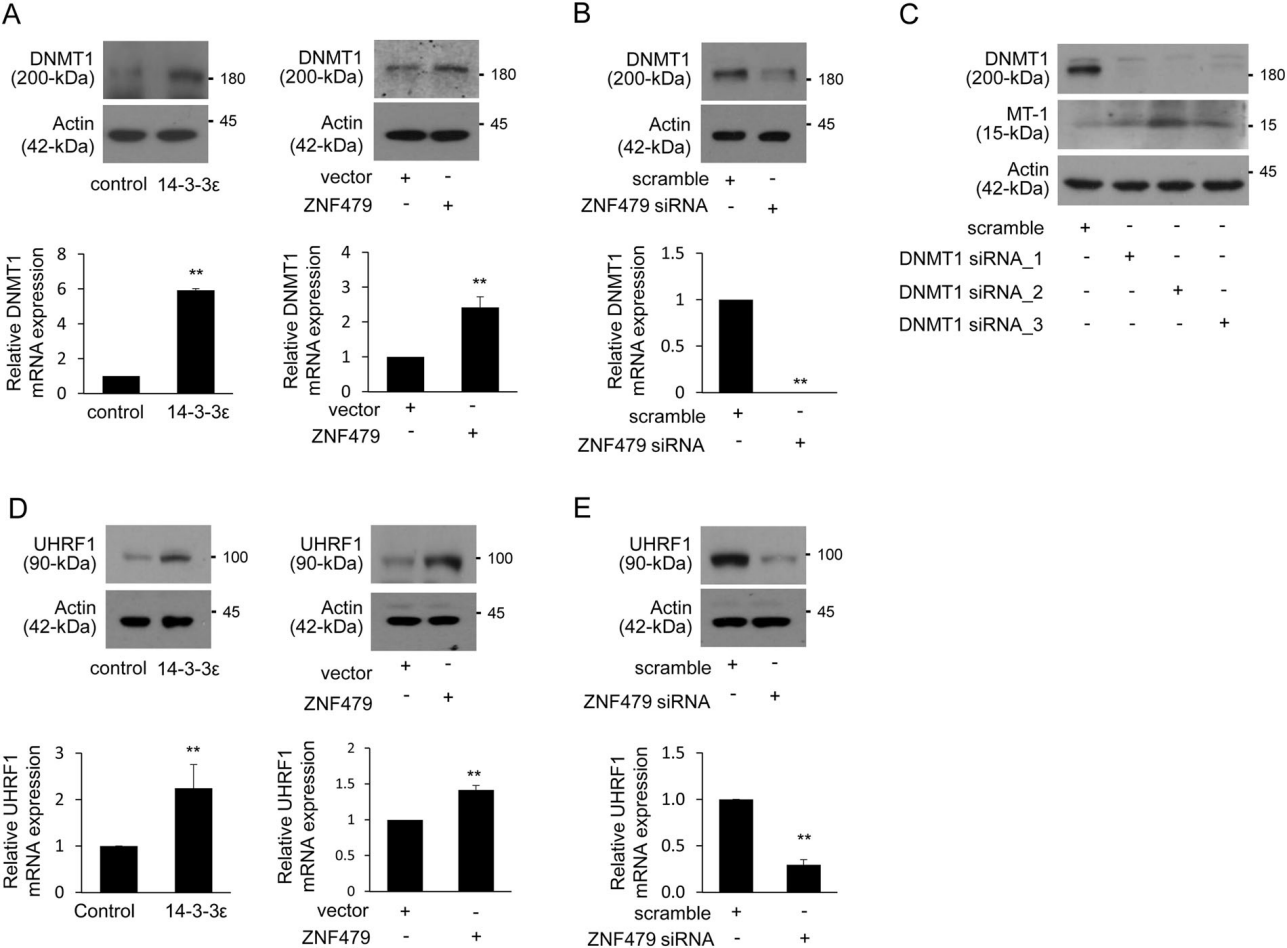
DNMT1 contributes to ZNF479-suppressed MT-1 expression. **a**, **b**, **d**, **e** Expression of DNMT1 and UHRF1 was examined by western blot and qPCR analysis in control/14-3-3ε–overexpressing cells (**a**, **d**, left), vector/ZNF479-transfected cells (right) and scramble/ZNF479 siRNA-transfected cells (**b**, **e**). Scale bars: mean ± SD. **P < 0.01. **c** Huh-7 cells were transfected with DNMT1 siRNA for 48 h. Expression of DNMT1 and MT-1 was determined by western blot analysis. Gene expression of qPCR analysis was normalized to both control cells as well as *GAPDH*. Actin was used as the loading control for western blotting analysis

The expression of MT-1 proteins is potentially repressed by DNMT1-mediated epigenetic regulation in HCC^19^. DNMT1 expression in 14-3-3ε- and ZNF479-overexpressing cells was increased (Fig. 4a), and ZNF479 siRNA reduced DNMT1 expression (Fig. 4b). siRNA silencing of DNMT1 significantly induced MT-1 expression (Fig. 4c). 14-3-3ε and ZNF479 overexpression induced UHRF1 expression (Fig. 4d), and ZNF479 siRNA significantly reduced UHRF1 expression (Fig. 4e).

### Expression of ZNF479, MT-1, ASH2L, Menin, DNMT1, and UHRF1 in PDTC-treated tumor-bearing mice and HCC

Because PDTC reduced in vivo tumor growth in nude mice (Fig. 1i), we examined the expression of ZNF479, MT-1, ASH2L, Menin, DNMT1, and UHRF1 in PDTC-treated tumors from mice (Fig. S10a). The expression of ZNF479, ASH2L, Menin, DNMT1, and UHRF1 was significantly reduced in both 50- and 100-mg/kg PDTC-treated tumors (Fig. S10b), but MT-1 expression was induced only in 100-mg/kg PDTC-treated tumors (Fig. S10b).

**Fig. 5:**
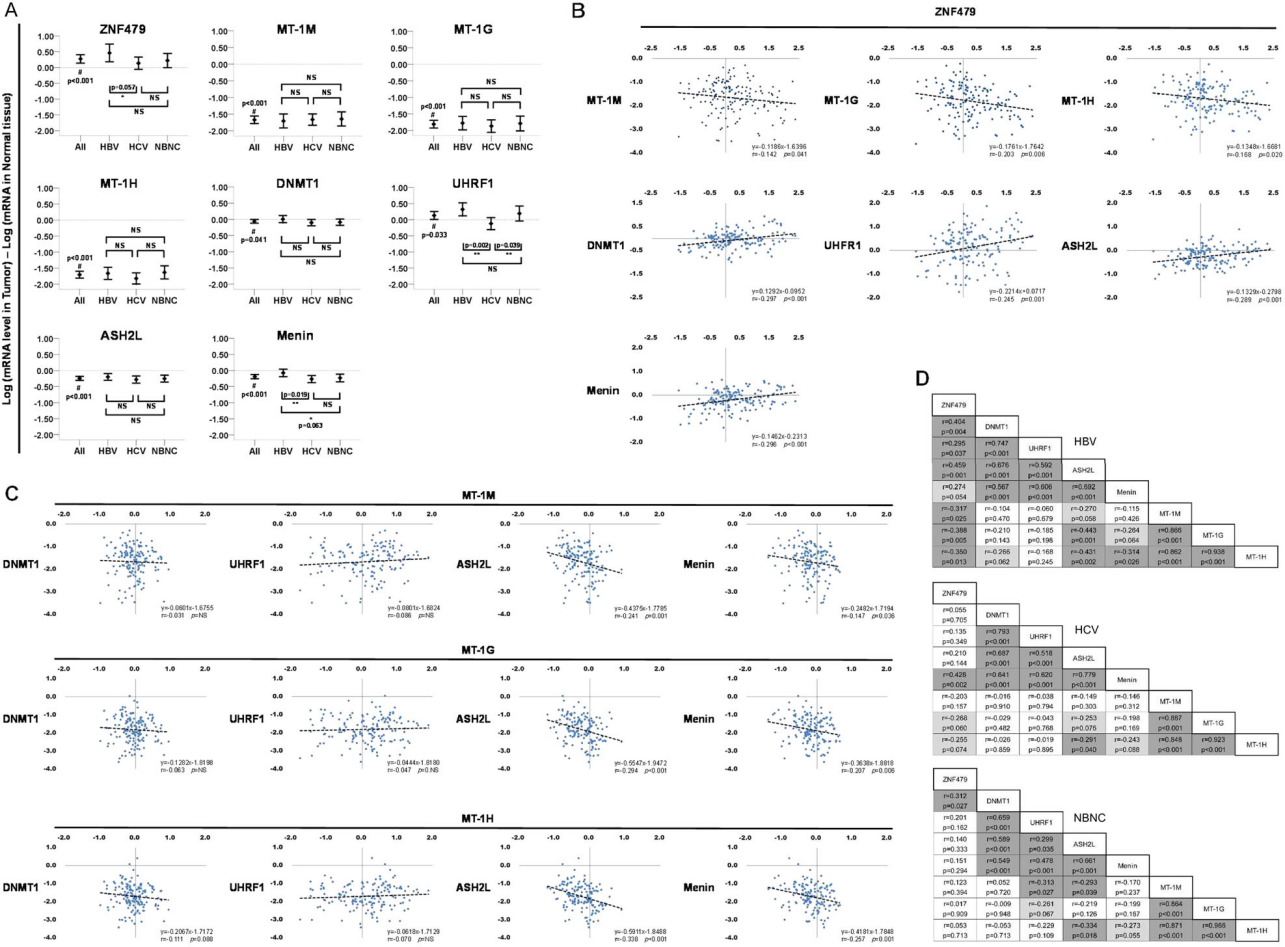
Expression analyses of ZNF479, MT-1M, MT-1G, MT-1H, ASH2L, Menin, DNMT1, and UHRF1 in HCC patients with HBV, HCV, or NBNC. **a** Expression of ZNF479, MT-1M, MT-1G, MT-1H, ASH2L, Menin, DNMT1, and UHRF1 in HCC tumors and paired normal liver tissues were analyzed by qPCR. Changes in expression were determined by Log (mRNA level in tumors)–Log (mRNA level in normal tissues). **b** Correlations between the expression of ZNF479 and MT-1M, MT-1G, MT-1H, ASH2L, Menin, DNMT1, and UHRF1 in HCC tissues. **c** Correlations between the expression of MT-1M, MT-1G, and MT-1H with ASH2L, Menin, DNMT1, and UHRF1 in HCC tissues. **d** Correlations between the expression of ZNF479, DNMT1, UHRF1, ASH2L, Menin, MT-1M, MT-1G, and MT-1H in clinical HCC samples were more prominent in patients in the HBV group than HCV and NBNC groups. Gene expression of qPCR analysis was normalized to both control cells as well as *GAPDH*

To further investigate the expression of ZNF479 and its regulated factors, we examined changes in expression of ZNF479, DNMT1, UHRF1, ASH2L, Menin, MT-1M, MT-1G, and MT-1H in a clinical HCC cohort (including 50 HBV, 50 HCV, and 50 NBNC) samples. We confirmed that the expression of MT-1 isoforms were decreased in HCC tumors compared to normal tissues (Fig. 5a). The expression of ZNF479 was significantly higher in HCC than that in the normal tissues (Fig. 5a). Unexpectedly, the overall expression of DNMT1, UHRF1, ASH2L, and Menin was lower in HCC than that in the normal tissues (Fig. 5a). Intriguingly, we found a positive correlation between the expression of ZNF479 and DNMT1, UHRF1, ASH2L, and Menin, but an inverse correlation with MT-1M, MT-1G, and MT-1H when analyzed in individual HCC samples (Fig. 5b). The expression of MT-1 isoforms was inversely correlated with ASH2L and Menin, but not significantly correlated with DNMT1 or UHRF1 (Fig. 5c). Finally, we found that the correlations with ZNF479 expression were more prominent in HCC patients in the HBV group than in the HCV and NBNC groups (Fig. 5d).

To further elucidate the expression correlation in patients of HBV group, we examined the protein levels of UHRF1, DNMT1, ASH2L, Menin, ZNF479, and MT-1 by western blotting analysis in HCC (29 samples) with HBV (Fig. S11). The relative protein levels (normalized with GAPDH) of ZNF479, ASH2L, UHRF1, and Menin were significantly higher in HCC than that in the normal tissues (Fig. S12). Although it is not statistically significant, there is a trend that protein levels of DNMT1 was higher but MT-1 was lower in HCC than normal tissues (Fig. S12).

## Discussion

In this study, we found that 14-3-3ε overexpression reduced the expression of MT-1 isoforms. Unexpectedly, siRNA knockdown of 14-3-3ε did not restore MT-1 expression, perhaps due to compensation by other 14-3-3 isoforms (Fig. S2). Inhibition of 14-3-3-ligand interactions by difopein significantly induced MT-1 expression (Fig. S3). Here, we obtained evidence that expression of ZNF479 is upregulated by 14-3-3ε overexpression (Fig. 2a) but suppressed by treatment with rapamycin, LY294002, and U0126 (Fig. 2c). As 14-3-3 proteins activate RAF/MEK/ERK, PI3K/Akt, and mTOR signaling^37^, ZNF479 expression may be induced by these pathways.

The expression of ASH2L is required for regulating H3K4 methylation, and siRNA knockdown of ASH2L reduces the level of H3K4me3^13^. Results from an earlier study indicated that ASH2L is expressed in 84.8% of HCC cases, but high levels of H3K4me2 are rarely found^38^. These results further confirm that ASH2L expression contributes to the modification of histone methylation in HCC. It has been reported that eliminating ASH2L reduces H3K4me3 but induces H3K4me2 levels^11^. In this study, we found that expression of MT-1 was inversely correlated with H3K4me3. We further examined the binding capacity of H3K4me3 on the MT-1 promoter in ZNF479-overexpressing cells by ChIP assay. We found that overexpression of ZNF479 only slightly influenced the binding capacity of H3K4me2 on the promoters of MT-1M, MT-1G, and MT-1H (Fig. S13). These results suggest that H3K4me3 modulates MT1 expression may mediate through an indirect regulation.

The MLL complex has methyltransferase activity and interacts with histone deacetylases and polycomb proteins, thereby suppressing the expression of HOX genes^39^. SUMO-specific isopeptidase is associated with the MLL complex, enhances the recruitment of ASH2L and Menin to the complex, and consequently turns on the transcriptional activity of HOX genes^40^. In this study, we show for the first time that ASH2L and Menin are upregulated by the 14-3-3ε/ZNF479 axis (Fig. 3b–e). As well, siRNA knockdown of ASH2L significantly induces MT-1 expression (Fig. 3f). MT-1 may be epigenetically suppressed by ZNF479 by inducing ASH2L expression. Further investigation is needed to elucidate how MT-1 is regulated by ASH2L.

Methylation of histone at the lysine residues plays a crucial role in the epigenetic regulation of gene expression. Although converted expression of H3K4me2 and H3K4me3 are modulated by ASH2L, both of H3K4me2 and H3K4me3 are considered as active factors in the promoter regions^41,42^. In addition to the methylation of H3K4, the symmetric di-methylation of histone H3 at arginine 2 (H3R2) was identified as a histone marker that supports euchromatin maintenance^41^. Results from several studies have indicated that the regulation of H3R2 and H3K4 methylation is mutually exclusive^42,43^. Here, we examined whether the expression of 14-3-3ε and ZNF479 affect di-methylation of H3R2 (H3R2me2). H3R2me2 was downregulated in 14-3-3ε- and ZNF479-overexpressing cells (Fig. S14a and S14b). In contrast, ZNF479 siRNA significantly induced H3R2me2 (Fig. S14c). Our findings of the regulation of histone H3 methylation echo the previous hypothesis of antagonism between ASH2L-regulated H3K4 and H3R2 methylation^42,43^.

KRAB-ZFPs regulate epigenetic modification by recruiting KAP-1. KAP-1 acts as a scaffold protein in the silencing complex consisting of histone methyltransferase SETDP1, nucleosome remodeling and deacetylation proteins, heterochromatin protein 1, and DNMTs, thereby impairing transcriptional activation^27^. Here we found that ZNF479 induces ASH2L, Menin, DNMT1, and UHRF1 expression (Figs. 3 and 4). Since ZNF479 contains a KRAB domain that is mostly known to induce transcriptional repression, it seems likely that this effect is indirect. How ZNF479 regulates the expression of DNMT1, UHRF1, ASH2L, Menin, or other MLL-complex proteins remains unclear and needs further investigation.

An earlier study demonstrated that PDTC synergizes with diethyl dithiocarbamate to augment MT-1 expression^44^. Here, we found that PDTC abundantly induced MT-1 expression (Fig. 1g) via an NFκB-independent mechanism (Fig. 1j). We identified that ZNF479 is a potential transcriptional regulator that is downregulated by PDTC (Fig. 2b and Table S1). To investigate whether MT-1 has a role in regulating ZNF479 level through negative feedback, we performed experiments of MT-1 overexpression and examined the expression level of ZNF479. Overexpression of MT-1M, MT-1G, and MT-1H had no significant effect on modulating ZNF479 expression (Fig. S15). Thus, the molecular regulation remains unclear and further investigation is needed to elucidate how PDTC suppresses ZNF479 expression.

The function and expression of ZNF479 have never been investigated. Here, we found that the expression level of ZNF479 was significantly higher in HCC (Fig. 5a). Our clinical analysis revealed that expression changes of ZNF479 were positively correlated with DNMT1, UHFR1, ASH2L, and Menin, but negatively correlate with MT-1 (Fig. 5b). Moreover, the expression of MT-1 isoforms was significantly negatively correlated with ASH2L and Menin (Fig. 5c). These results suggest that MLL complex has a more predominant role in regulating MT-1 expression. Interestingly, the correlated expression of ZNF479 with other downstream factors is more prominent in the HBV positive group (Fig. 5d). These results suggest that ASH2L and Menin play an important role in ZNF479-downregulated MT-1 expression. Further investigation is needed to elucidate the roles of ZNF479 and the MLL complex in HCC tumorigenesis and tumor progression.

**Fig. 6:**
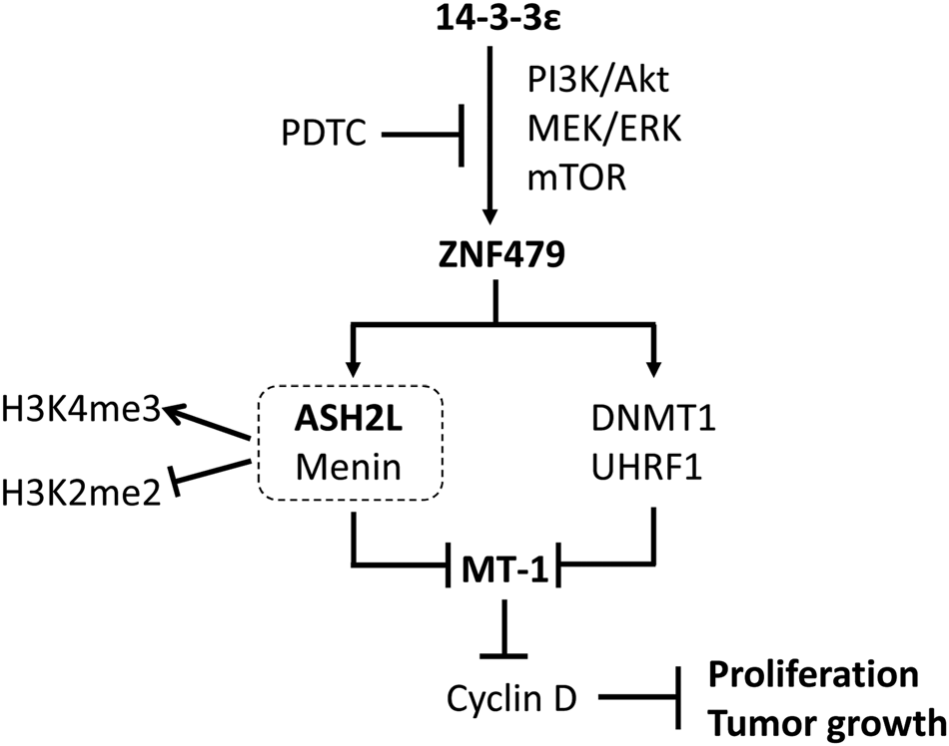
Proposed model in this study for how ZNF479 suppresses MT-1 expression by regulating ASH2L and DNMT1 in HCC

In this study, we found that MT-1 was suppressed by 14-3-3ε, and overexpression of MT-1 abolished 14-3-3ε-induced HCC cell proliferation, and tumor growth. We identified that ZNF479 is involved in 14-3-3ε-suppressed MT-1 expression by upregulating DNMT1, UHRF1, ASH2L, and Menin (illustrated scheme summarized in in Fig. 6). We have further confirmed the expression results of ZNF479, MT-1, ASH2L, Menin, DNMT1, UHRF1, H3K4me2, and H3K4me3 from Huh-7 cells in HepG2 and Hep3B (Fig. S16). Developing approaches targeting 14-3-3ε, ZNF479, MT-1, MLL-complex components, and related factors may be a potential therapeutic strategy for HCC.

## Materials and methods

### Cell culture and reagents

The cell lines Huh-7 (Japanese Collection of Research Bioresources, JCRB-0403) and HepG2 (American Type Culture Collection, ATCC-HB-8065) were cultured in Dulbecco’s modified Eagle medium (DMEM, Gibco, Gaithersburg, MD, USA) supplemented with 10% FBS (Hyclone, Thermo Fisher Scientific, Waltham, MA, USA), 100 U/ml penicillin, 100 mg/ml streptomycin at 37℃ in a humidified 5% CO_2_ atmosphere. A stable Huh-7 cell line, which overexpresses FLAG-tagged 14-3-3ε, was previously established and maintained in DMEM with 10% FBS and 200 μg/ml G418^36^. All pharmacological inhibitors were purchased from Sigma–Aldrich (St. Louis, MO, USA). Recombinant human DKK-1 was purchased from PeproTech (Rocky Hill, NJ, USA).

### Plasmids, siRNAs, and transfection

The full length coding sequences of human 14-3-3ε, ZNF479, and MT-1 isoforms (MT-1M, MT-1G, and MT1H) were amplified by PCR and cloned into p3XFLAG-CMV and pcDNA3.1/*myc*-His(-)-A vectors (primer sequences in Table S2). The sequences of 14-3-3 antagonist difopein oligonucleotides (sequences in Table S2)^45^ were synthesized (MDBio, Inc.) and cloned into the pcDNA3.1/*myc*-His(-)-A vector. Expression vectors were transfected into cells using PolyJet in vitro DNA transfection reagent (SignaGen Laboratories, Gaithersburg, MD, USA). Transfection of small interfering RNA (siRNA, sequences listed in Table S3) for ZNF479, DNMT1, ASH2L (Shanghai GenePharma, China), and 14-3-3ɛ (Stealth RNAi, Invitrogen, Carlsbad, CA) involved using Lipofectamine RNAiMAX (ThermoFisher Scientific, Victoria, Australia).

### Western blot analysis

Protein expression was analyzed by western blotting analysis described previously^36^. In brief, cells were harvested and protein concentrations were determined using a Bio-Rad Protein Assay Kit (Bio-Rad Laboratories, Hercules, CA, USA). Lysates (30 µg) were separated by SDS-PAGE, then the resolved proteins were transferred to PVDF membranes (Millipore, Bedford, MA, USA). Incubation with primary antibodies (listed in Table S4) was performed overnight at 4 ℃. After washing with PBS-T, membranes were incubated with horseradish peroxidase-conjugated secondary antibodies. The protein signals were determined by enhanced chemiluminescence (PerkinElmer, Shelton, CT). Quantification of protein expression was determined by western blot and quantified by ImageJ 1.43 u software

### Cell proliferation analysis and anchorage-independent growth assay

Cell proliferation was measured using an MTT assay (Sigma–Aldrich, USA). Absorbance at 570 nm was measured with a reference wavelength of 690 nm. Anchorage-independent growth was assessed by a soft-agar assay. Six-well plates were coated with 2 ml 10% complete medium containing 0.8% low-melting agarose (Lonza, Rockland, ME), then seeded with 5 × 10^3^ cells per well. After 3 weeks, cells were stained with crystal violet and colony numbers were counted.

### Quantitative real-time PCR (qPCR)

Total RNA was extracted using RNAzol RT (RN190, Molecular Research Center, USA) and cDNAs were synthesized using the PrimeScript RT reagent kit (TAKARA, Japan). qPCR analysis involved use of SYBR Green (Kapabiosystem, Woburn, MA, USA) after probing with specific oligonucleotide primers (Table S5). Applied Biosystems Relative Quantification Manager Software v1.2 was used to analyze the relative gene expression by the comparative cycle threshold (Ct) method. Gene expression was normalized to that of glyceraldehyde-3-phosphate dehydrogenase (*GAPDH*).

### Tumor xenograft experiments

The protocol of this study was performed in accordance with the guidelines and regulations approved by the Institutional Animal Care and Use Committee of the National Health Research Institutes. Briefly, 8-week-old BALB/c nu/nu nude mice were purchased from the National Laboratory Animal Center, National Health Research Institutes of Taiwan and housed in microisolator cages in a specific pathogen-free facility. A total of 2 × 10^6^ 14-3-3ε overexpression cells or 4 × 10^6^ *MT-1M* transfected cells were subcutaneously injected into the right flank of nude mice^32^. For pyrrolidine dithiocarbamate (PDTC) treatment, mice (that received 2 × 10^6^ 14-3-3ε cells for 1 week) were peri-tumorally injected with vehicle control (PBS) or PDTC (50 and 100 mg/kg)^46^ every 2 days for 28 days. The tumor volume was determined by sequential caliper measurements of length (L) and width (W) and calculated as LW^2^/2. Mice were sacrificed and tumor weight was measured after cultivation for 5 weeks.

### Small-hairpin RNA (shRNA) xenograft experiment

pLKO-TRC005–derived ZNF479 small-hairpin RNAs (shRNA) were purchased from the National RNAi Core Facility, Taiwan (target sequences listed in Table S6). shRNAs were transfected into Huh-7 cells, then stabilized with 2 μg/ml puromycin. The knockdown efficiency of the shRNAs was examined by western blot analysis of ZNF479 expression. For the tumor xenograft model, 2 × 10^6^ stable cells (ZNF479 shRNA: TRCN0000239328; control: ASN0000000003) were injected into 8-week-old nude mice. Tumor volume was determined every week for 5 weeks, and tumor weight was measured after mice were sacrificed.

### Microarray analysis

Gene expression profile with PDTC treatment in 14-3-3ε–overexpressing stable cells was examined by microarray analysis. RNA samples were analyzed using the Affymetrix Human Gene 1.0 ST array (Affymetrix Inc., Santa Clara, CA, USA) according to the manufacturer’s recommendations.

### Clinical specimens

mRNA expression levels were assessed in 300 tissue RNA extractions (including normal liver and HCC) from HCC patients. Patient samples were divided into three groups: 50 HBV, Hepatitis B; 50 HCV, Hepatitis C; and 50 NBNC, neither Hepatitis B nor C. Expression levels of ZNF479, DNMT1, UHRF1, ASH2L, Menin, MT-1M, MT-1G, and MT-1H were examined by qPCR analysis.

### Chromatin immunoprecipitation (ChIP)

Interaction of H3K4me3 in the *MT-1* promoters was examined using Chromatin Immunoprecipitation Kits (17-10086, Millipore, CA, USA). Briefly, 2 × 10^6^ cells were used for each ChIP. DNA-protein complex was cross-linked with 1% formaldehyde (Sigma–Aldrich, USA) for 10 min and then washed with PBS. Quenched cells with 1.25 mM Glycine for 5 min. Cells were collected and lysed in lysis buffer containing protease inhibitor cocktail (Sigma–Aldrich, USA). Cross-linked DNA was sheared to 1000–200 bp in length and immunoprecipitated with 1 μg of anti-H3K4me3 or normal rabbit IgG in 4 ℃ for overnight. Proteins were degraded by proteinase K and genomic DNA was purified using spin columns and eluted in 50 μl of water. Primer sequences used for PCR were listed in Table S7.

### Statistical analysis

For clinical samples, changes in gene expression in tumors were determined by assessing the differences between normal and tumor tissues, calculated through the log of mRNA expression in tumor tissue minus normal tissue. These results were then analyzed by a one-sample Student *t*-test with zero as the test value. A one-way ANOVA test was used when comparing gene expression changes in tumors between different patient groups. To compare expression levels of different genes in the tumors, a Pearson’s correlation co-efficient (r) was calculated, and a linear regression formula was provided if 2-axis dot plots were drawn. Two-side *p* < 0.05 is considered as statistically significant. All statistical analyses were performed with SPSS software (version 17.0).

## Supplementary information

Supplementary materials

## References

[1] Coyle P, Philcox JC, Carey LC, Rofe AM (2002). Metallothionein: the multipurpose protein. Cell. Mol. life Sci..

[2] West AK (1990). Human metallothionein genes: structure of the functional locus at 16q13. Genomics.

[3] Pedersen MO, Larsen A, Stoltenberg M, Penkowa M (2009). The role of metallothionein in oncogenesis and cancer prognosis. Prog. Histochem. Cytochem..

[4] Huang GW, Yang LY (2002). Metallothionein expression in hepatocellular carcinoma. World J. Gastroenterol..

[5] Datta J (2007). Metallothionein expression is suppressed in primary human hepatocellular carcinomas and is mediated through inactivation of CCAAT/enhancer binding protein alpha by phosphatidylinositol 3-kinase signaling cascade. Cancer Res..

[6] Ji XF (2014). MT1M and MT1G promoter methylation as biomarkers for hepatocellular carcinoma. World J. Gastroenterol..

[7] Mao J (2012). Metallothionein MT1M is a tumor suppressor of human hepatocellular carcinomas. Carcinogenesis.

[8] Yokoyama A (2004). Leukemia proto-oncoprotein MLL forms a SET1-like histone methyltransferase complex with menin to regulate Hox gene expression. Mol. Cell. Biol..

[9] Luscher-Firzlaff J (2008). The human trithorax protein hASH2 functions as an oncoprotein. Cancer Res..

[10] Southall SM, Wong PS, Odho Z, Roe SM, Wilson JR (2009). Structural basis for the requirement of additional factors for MLL1 SET domain activity and recognition of epigenetic marks. Mol. Cell.

[11] Steward MM (2006). Molecular regulation of H3K4 trimethylation by ASH2L, a shared subunit of MLL complexes. Nat. Struct. Mol. Biol..

[12] Hughes CM (2004). Menin associates with a trithorax family histone methyltransferase complex and with the hoxc8 locus. Mol. Cell.

[13] Xu B (2013). Menin promotes hepatocellular carcinogenesis and epigenetically up-regulates Yap1 transcription. Proc. Natl Acad. Sci. USA.

[14] Zindy PJ (2006). Upregulation of the tumor suppressor gene menin in hepatocellular carcinomas and its significance in fibrogenesis. Hepatology.

[15] Choi MS (2003). Expression of DNA methyltransferases in multistep hepatocarcinogenesis. Hum. Pathol..

[16] Saito Y (2003). Increased protein expression of DNA methyltransferase (DNMT) 1 is significantly correlated with the malignant potential and poor prognosis of human hepatocellular carcinomas. Int. J. Cancer.

[17] Ghoshal K (2002). Inhibitors of histone deacetylase and DNA methyltransferase synergistically activate the methylated metallothionein I promoter by activating the transcription factor MTF-1 and forming an open chromatin structure. Mol. Cell. Biol..

[18] Majumder S (2006). Epigenetic regulation of metallothionein-i gene expression: differential regulation of methylated and unmethylated promoters by DNA methyltransferases and methyl CpG binding proteins. J. Cell. Biochem..

[19] Takata A (2013). MicroRNA-140 acts as a liver tumor suppressor by controlling NF-kappaB activity by directly targeting DNA methyltransferase 1 (Dnmt1) expression. Hepatology.

[20] Bostick M (2007). UHRF1 plays a role in maintaining DNA methylation in mammalian cells. Science.

[21] Rothbart SB (2012). Association of UHRF1 with methylated H3K9 directs the maintenance of DNA methylation. Nat. Struct. Mol. Biol..

[22] Sharif J (2007). The SRA protein Np95 mediates epigenetic inheritance by recruiting Dnmt1 to methylated DNA. Nature.

[23] Arita K, Ariyoshi M, Tochio H, Nakamura Y, Shirakawa M (2008). Recognition of hemi-methylated DNA by the SRA protein UHRF1 by a base-flipping mechanism. Nature.

[24] Liu X (2017). Elevated UHRF1 expression contributes to poor prognosis by promoting cell proliferation and metastasis in hepatocellular carcinoma. Oncotarget.

[25] Margolin JF (1994). Kruppel-associated boxes are potent transcriptional repression domains. Proc. Natl Acad. Sci. USA.

[26] Urrutia R (2003). KRAB-containing zinc-finger repressor proteins. Genome Biol..

[27] Ecco, G., Imbeault, M. & Trono, D. KRAB zinc finger proteins. **144**, 2719–2729, 10.1242/dev.132605 (2017). 10.1242/dev.132605 PMC7117961 28765213

[28] Friedman JR (1996). KAP-1, a novel corepressor for the highly conserved KRAB repression domain. Genes Dev..

[29] Mark C, Looman C, Abrink M, Hellman L (2001). Molecular cloning and preliminary functional analysis of two novel human KRAB zinc finger proteins, HKr18 and HKr19. DNA Cell Biol..

[30] Darling DL, Yingling J, Wynshaw-Boris A (2005). Role of 14-3-3 proteins in eukaryotic signaling and development. Curr. Top. Dev. Biol..

[31] Ko BS (2011). Overexpression of 14-3-3epsilon predicts tumour metastasis and poor survival in hepatocellular carcinoma. Histopathology.

[32] Liu TA (2011). Increased expression of 14-3-3beta promotes tumor progression and predicts extrahepatic metastasis and worse survival in hepatocellular carcinoma. Am. J. Pathol..

[33] Ko BS (2011). Involvement of 14-3-3gamma overexpression in extrahepatic metastasis of hepatocellular carcinoma. Hum. Pathol..

[34] Huang XY (2013). alphaB-crystallin complexes with 14-3-3zeta to induce epithelial-mesenchymal transition and resistance to sorafenib in hepatocellular carcinoma. Hepatology.

[35] Liu CC (2014). 14-3-3sigma induces heat shock protein 70 expression in hepatocellular carcinoma. BMC Cancer.

[36] Liu TA (2013). 14-3-3epsilon overexpression contributes to epithelial-mesenchymal transition of hepatocellular carcinoma. PloS ONE.

[37] Wu, Y. J., Ko, B. S. & Liou, J. Y. in *Encyclopedia of Signaling Molecules* (ed S. Choi) 1–11 (Springer Nature, Switzerland, 2018).

[38] Magerl C (2010). H3K4 dimethylation in hepatocellular carcinoma is rare compared with other hepatobiliary and gastrointestinal carcinomas and correlates with expression of the methylase Ash2 and the demethylase LSD1. Hum. Pathol..

[39] Xia ZB, Anderson M, Diaz MO, Zeleznik-Le NJ (2003). MLL repression domain interacts with histone deacetylases, the polycomb group proteins HPC2 and BMI-1, and the corepressor C-terminal-binding protein. Proc. Natl Acad. Sci. USA.

[40] Nayak A, Viale-Bouroncle S, Morsczeck C, Muller S (2014). The SUMO-specific isopeptidase SENP3 regulates MLL1/MLL2 methyltransferase complexes and controls osteogenic differentiation. Mol. Cell.

[41] Migliori V (2012). Symmetric dimethylation of H3R2 is a newly identified histone mark that supports euchromatin maintenance. Nat. Struct. Mol. Biol..

[42] Guccione E (2007). Methylation of histone H3R2 by PRMT6 and H3K4 by an MLL complex are mutually exclusive. Nature.

[43] Kirmizis A (2007). Arginine methylation at histone H3R2 controls deposition of H3K4 trimethylation. Nature.

[44] Ren Y, Smith A (1995). Mechanism of metallothionein gene regulation by heme-hemopexin. Roles of protein kinase C, reactive oxygen species, and cis-acting elements. J. Biol. Chem..

[45] Masters SC, Fu H (2001). 14-3-3 proteins mediate an essential anti-apoptotic signal. J. Biol. Chem..

[46] Liu LP (2010). The role of NF-kappaB in Hepatitis b virus X protein-mediated upregulation of VEGF and MMPs. Cancer Invest..

