# Supplementary material for: ZNF479 downregulates metallothionein-1 expression by regulating ASH2L and DNMT1 in hepatocellular carcinoma
Source: Cell Death Dis. 2019 May 28;10(6):408. doi: 10.1038/s41419-019-1651-9 (PMC6538656; doi:10.1038/s41419-019-1651-9)
Supplement: Supplementary file 1 — Supplementary materials [file 41419_2019_1651_MOESM1_ESM.docx]

**Supporting information**

**Table S1.** PDTC induced or reduced genes analyzed by microarray (Affymetrix Human Gene 1.0 ST array) in 14-3-3ε cells.

| **Increase** | | | | | | **Decrease** | | | | |
| --- | --- | --- | --- | --- | --- | --- | --- | --- | --- | --- |
| **RefSeq** | **Gene**  **Symbol** | **Fold** | **RefSeq** | **Gene**  **Symbol** | **Fold** | **RefSeq** | **Gene Symbol** | **Fold** | |  |
| NM_005950 | MT1G | 22.41 | NM_020368 | UTP3 | 2.37 | NM_001098408 | GAGE12C | | -4.67 | |
| NM_005947 | MT1B | 22.06 | NM_014278 | HSPA4L | 2.36 | AK126788 | FLJ44838 | | -4.66 | |
| NM_005953 | MT2A | 12.6 | NM_002155 | HSPA6 | 2.35 | NM_001105662 | USP17 | | -3.32 | |
| NM_005951 | MT1H | 11.93 | NM_001713 | BHMT | 2.35 | NM_001135599 | TGFB2 | | -3.27 | |
| NM_005952 | MT1X | 10.64 | NM_002631 | PGD | 2.29 | NR_027279 | USP17L6P | | -3.14 | |
| NM_176870 | MT1M | 10.24 | NM_021030 | ZNF14 | 2.28 | NM_001137671 | POTEC | | -3.05 | |
| NM_005949 | MT1F | 7.97 | NM_024640 | YRDC | 2.28 | NM_001142800 | EYS | | -2.72 | |
| NM_005946 | MT1A | 6.7 | NM_176884 | TAS2R43 | 2.27 | NM_001001824 | OR2T27 | | -2.72 | |
| NM_032717 | AGPAT9 | 5.93 | NM_203301 | FBXO33 | 2.25 | NM_173512 | SLC38A11 | | -2.67 | |
| NR_001447 | MT1L | 5.9 | NM_001074 | UGT2B7 | 2.23 | NM_001099694 | ZNF578 | | -2.64 | |
| NM_020299 | AKR1B10 | 5.8 | NM_021029 | RPL36A | 2.22 | NM_002171 | IFNA10 | | -2.5 | |
| NM_000499 | CYP1A1 | 5.1 | NM_000559 | HBG1 | 2.22 | ENST00000331301 | FLJ42102 | | -2.48 | |
| NR_027781 | MT1DP | 4.85 | NM_000184 | HBG2 | 2.22 | NM_001098406 | GAGE12J | | -2.46 | |
| NR_003669 | MT1IP | 4.75 | NM_032689 | ZNF607 | 2.2 | AF284768 | RPSAP15 | | -2.45 | |
| NM_002150 | HPD | 4.41 | NM_002061 | GCLM | 2.19 | AK300495 | CWH43 | | -2.44 | |
| NM_003937 | KYNU | 4.29 | NM_032890 | DISP1 | 2.17 | NM_201402 | USP17L2 | | -2.43 | |
| AF348994 | MT1JP | 4.07 | NM_000169 | GLA | 2.17 | XM_001714030 | LOC642838 | | -2.42 | |
| NR_003321 | SNORD116-6 | 4.04 | NM_001748 | CAPN2 | 2.17 | NM_207299 | LPPR1 | | -2.39 | |
| NM_021187 | CYP4F11 | 3.77 | NM_005635 | SSX1 | 2.17 | NM_139173 | NHEDC1 | | -2.39 | |
| BC133653 | MT1P3 | 3.7 | NM_001010844 | IRAK1BP1 | 2.16 | NR_003594 | REXO1L2P | | -2.38 | |
| NR_015379 | UCA1 | 3.36 | NR_026750 | DKFZp686O24166 | 2.13 | AF495523 | REXO1L1 | | -2.38 | |
| NM_001885 | CRYAB | 3.33 | NM_014344 | FJX1 | 2.12 | NM_000765 | CYP3A7 | | -2.37 | |
| NM_015187 | SEL1L3 | 3.19 | NM_003527 | HIST1H2BO | 2.12 | ENST00000318245 | FLJ35379 | | -2.37 | |
| NM_001373 | DNAH14 | 3.17 | BC126397 | C2orf76 | 2.11 | NM_006017 | PROM1 | | -2.35 | |
| NM_000584 | IL8 | 3.1 | NM_005058 | RBMY1A1 | 2.11 | NM_003657 | BCAS1 | | -2.34 | |
| NM_005909 | MAP1B | 3.08 | NM_004477 | FRG1 | 2.11 | NM_001004705 | OR4D10 | | -2.31 | |
| NM_014331 | SLC7A11 | 3.07 | NR_002190 | SUMO1P3 | 2.09 | NM_001964 | EGR1 | | -2.31 | |
| NM_175617 | MT1E | 3.05 | NM_016315 | GULP1 | 2.09 | NM_004172 | SLC1A3 | | -2.31 | |
| NM_003309 | TSPYL1 | 2.85 | NM_006959 | ZNF17 | 2.09 | NR_027320 | CSNK1A1P | | -2.3 | |
| NM_001137550 | LRRFIP1 | 2.75 | NM_023039 | ANKRA2 | 2.08 | AF113887 | IGKC | | -2.29 | |
| NM_001113528 | METT5D1 | 2.74 | NM_006582 | GMEB1 | 2.08 | AF143328 | OR52K3P | | -2.29 | |
| NM_002133 | HMOX1 | 2.7 | BC040288 | LOC100130428 | 2.08 | AF161557 | HSPC072 | | -2.26 | |
| XR_079074 | LCN1L1 | 2.68 | NM_007189 | ABCF2 | 2.08 | NM_002170 | IFNA8 | | -2.22 | |
| NR_002907 | SNORA73A | 2.67 | NM_001866 | COX7B | 2.06 | NM_006449 | CDC42EP3 | | -2.22 | |
| NM_000433 | NCF2 | 2.66 | NM_032935 | MT4 | 2.06 | NM_033273 | ZNF479 | | -2.2 | |
| NM_018260 | ZNF701 | 2.66 | NR_003335 | SNORD116-21 | 2.06 | ENST00000316004 | OR4H12P | | -2.17 | |
| NM_022060 | ABHD4 | 2.64 | NM_001007537 | C1QTNF9B | 2.06 | NM_032681 | SPRYD5 | | -2.15 | |
| NM_005345 | HSPA1A | 2.63 | NM_001038640 | GOLGA6A | 2.04 | NR_027714 | FKSG73 | | -2.13 | |
| XM_001716780 | LOC648822 | 2.62 | NM_005320 | HIST1H1D | 2.04 | NR_026643 | FAM99A | | -2.13 | |
| NM_207366 | 41166 | 2.58 | AK097079 | LOC100129884 | 2.04 | NM_001005853 | OR6B2 | | -2.12 | |
| NM_002374 | MAP2 | 2.5 | NM_024644 | C14orf169 | 2.04 | NM_000354 | SERPINA7 | | -2.12 | |
| ENST00000456556 | LOC400986 | 2.49 | NM_138768 | MYEOV | 2.03 | NM_019893 | ASAH2 | | -2.11 | |
| NM_033390 | ZC3H12C | 2.45 | NM_014878 | KIAA0020 | 2.03 | NR_002141 | OR2M1P | | -2.11 | |
| AY956762 | HSP90AA6P | 2.44 | NM_001136501 | ZNF844 | 2.03 | NM_005492 | CST8 | | -2.1 | |
| NM_032681 | SPRYD5 | 2.43 | NM_001105539 | ZBTB10 | 2.03 | NM_207317 | ZNF474 | | -2.07 | |
| NM_000904 | NQO2 | 2.42 | NM_031412 | GABARAPL1 | 2.03 | NM_145203 | CSNK1A1L | | -2.07 | |
| NM_025130 | HKDC1 | 2.41 | NM_006980 | MTERF | 2.03 | NM_001455 | FOXO3 | | -2.06 | |
| NM_001902 | CTH | 2.4 | NM_021932 | RIC8A | 2.02 | NM_005666 | CFHR2 | | -2.06 | |
| NM_001143957 | GPR63 | 2.39 | NR_003336 | SNORD116-22 | 2.02 | ENST00000442886 | ZNF840P | | -2.05 | |
| NM_000700 | ANXA1 | 2.39 | NM_020765 | UBR4 | 2.02 | NM_024420 | PLA2G4A | | -2.05 | |
| NM_003986 | BBOX1 | 2.39 | NM_006729 | DIAPH2 | 2.01 | NR_003357 | SNORD115-42 | | -2.04 | |
| NR_003276 | CES4 | 2.38 | NM_016391 | NOP16 | 2.01 | NM_003122 | SPINK1 | | -2.04 | |
| NM_183421 | FBXO25 | 2.38 | NR_003521 | WHAMML1 | 2 | NR_029957 | MIR429 | | -2.01 | |

**Table S2.** Primer sequences for the plasmid construction.

| **Gene name** | **Primer sequences** |
| --- | --- |
| MT-1M | ATGGACCCCAACTGCTCCTG |
|  | GGCACAGCAGCTGCAGTTCT |
| MT-1G | ATGGACCCCAACTGCTCCTGTGCCGCTGGT |
|  | GGCGCAGCAGCTGCACTTCTCCGATGC |
| MT-1H | ATGGACCCCAACTGCTCCTGCGAGGCTGGT |
|  | GGCACAGCAGCTGCACTTCTCTGACGC |
| ZNF479 | ATGGCTAAAAGACCAGGA |
|  | TTCACATTTGTAGGGTTTCT |
|  | |
| **Oligonucleotides sequence** | |
| Difopein | CCTCATTGTGTTCCTAGAGATCTTAGTTGGCTTGATCTTGAAGCTAATATGTGTCTTCCTGGTGCTGCTGGTCTTGATAGTGCTGATGGTGCTCCTCATTGTGTTCCTAGAGATCTTAGTTGGCTTGATCTTGAAGCTAATATGTGTCTTCCT |

**Table S3.** Sequences of siRNAs used in this study.

| **Source** | **GenePharma** |
| --- | --- |
| **Name** | **sequence** |
| Negative Control | UUCUCCGAACGUGUCACGUTT |
|  | ACGUGACACGUUCGGAGAATT |
| ZNF479-homo-447 | UCUCUAAGCCAGACUUGAUTT |
|  | AUCAAGUCUGGCUUAGAGATT |
| ZNF479-homo-687 | GGAGGUUAUAGUGAAGUUATT |
|  | UAACUUCACUAUAACCUCCTT |
| ZNF479-homo-938 | CUGCUCCUCAAACCAUACUTT |
|  | AGUAUGGUUUGAGGAGCAGTT |
| DNMT1-homo-1665 | GCACCUCAUUUGCCGAAUATT |
|  | UAUUCGGCAAAUGAGGUGCTT |
| DNMT1-homo-2505 | GGGACUGUGUCUCUGUUAUTT |
|  | AUAACAGAGACACAGUCCCTT |
| DNMT1-homo-3485 | GAGGCCUAUAAUGCAAAGATT |
|  | UCUUUGCAUUAUAGGCCUCTT |
| ASH2L siRNA_1 ^1^ | CCGAGUAACUAACUUAUUUAATT |
|  | UUAAAUAAGUUAGUUACUCGGTT |
| AHS2L siRNA_2 ^1^ | CCCGUUUAACAAAGAUGGCUATT |
|  | UAGCCAUAUUUGUUAAACGGGTT |
| ASH2L-homo-1455 | CCAAGUUCCACCAGUCCAUTT |
|  | AUGGACUGGUGGAACUUGGTT |
|  |  |
| **Source** | **Invitrogen** |
| **Name** | **Sequence** |
| Negative Control GC duplex #2 | Cat.No.12935-112 sequence is not available |
| 14-3-3ε siRNA_1 | AAACCAUUACAACGAAGUCCCUCCC  GGGAGGGACUUCGUUGUAAUGGUUU |
| 14-3-3ε siRNA_2 | UUCUUCAACUGUCAGCUCCACAUCC  GGAUGUGGAGCUGACAGUUGAAGAA |
| 14-3-3ε siRNA_3 | UUAAGAAUUUCGUAGUAGAAUACGG  CCGUAUUCUACUACGAAAUUCUUAA |
|  | |

**Table S4.** Antibodies used in this study.

| **Antibody** | **cat. No.** | **Company** | **RRID** |
| --- | --- | --- | --- |
| 14-3-3ε | sc-1020 | Santa Cruz | AB_630821 |
| aceH3 | 06-599 | Millpore | AB_2115283 |
| aceH4 | 06-866 | Millpore | AB_310270 |
| AKT | #4685 | Cell signaling | AB_2225340 |
| ASH2L | 12331-1-AP | Proteintech | AB_2059837 |
| β-Catenin | 610153 | BD | AB_397554 |
| Cyclin D1 | #2922 | Cell signaling | AB_2228523 |
| DNMT1 | #5032 | Cell signaling | AB_10548197 |
| ERK1/2 | #9102 | Cell signaling | AB_330744 |
| Flag | F3165 | Sigma | AB_259529 |
| H3K4me2 | #9725 | Cell signaling | AB_10205451 |
| H3K4me3 | 07-473 | Millpore | AB_1977252 |
| H3R2me2 | GTX124114 | GeneTex | AB_2752252 |
| Histone H3 | GTX122148 | GeneTex | AB_10633308 |
| Histone H4 | 07-108 | Millipore | AB_2279758 |
| Menin | #6891 | Cell signaling | AB_10858216 |
| MT-1 | 18-0133 | Invitrogen | AB_138681 |
| p65 | sc-109 | Santa Cruz | AB_632039 |
| Phosphor-AKT  (Thr308)  Phosphor-AKT  (Ser473) | #2965  #4060 | Cell signaling  Cell signaling | AB_2255933  AB_2315049 |
| Phosphor-ERK1/2 | #9101 | Cell signaling | AB_331646 |
| ZNF479 | ab116305 | Abcam | AB_10899421 |

**Table S5.** Primer sequences for qPCR used in this study.

| **Gene name** | **qPCR primer sequence** |
| --- | --- |
| GAPDH | CGCTCTCTGCTCCTCCTGTT |
|  | CCATGGTGTCTGAGCGATGT |
| 14-3-3ε | AATGATTCGGGAATATCGGCAAA |
|  | ACTCGCCAGTGTTAGCTGC |
| MT1A | GCTTGGGATCTCCAACCTCAC |
|  | TTGCAGGAGGTGCATTTG |
| MT-1E ^2^ | GCTTGTTCGTCTCACTGGTG |
|  | CAGGTTGTGCAGGTTGTTCTA |
| MT-1F ^2^ | AGTCTCTCCTCGGCTTGC |
|  | ACATCTGGGAGAAAGGTTGTC |
| MT1G ^3^ | CTTCTCGCTTGGGAACTCTA |
|  | AGGGGTCAAGATTGTAGCAAA |
| MT1H | CCTCTTCTCTTCTCGCTTGG |
|  | TTGCAGGAGGTGCATTTG |
| MT-1M ^3^ | TCCGGGTGGGCCTAGCAGTCG |
|  | AATGCAGCAAATGGCTCAGTATCGTATTG |
| MT-1X ^3^ | TCTCCTTGCCTCGAAATGGAC |
|  | GGGCACACTTGGCACAGC |
| ZNF479 | GTCCCAGAATATAAAGAGAAATGAG |
|  | GGGTAGTTGACAAACATTGG |
| DNMT1 | AGAACGGTGCTCATGCTTACA |
|  | CTCTACGGGCTTCACTTCTTG |
| ASH2L | ATGGCAGTCCCGAACACAG |
|  | GGTTGTCATGCACTCCCAGTAT |
| UHRF1 ^4^ | GCCATACCCTCTTCGACTACG |
|  | GCCCCAATTCCGTCTCATCC |
| Menin ^5^ | ATCACAGGCACCAAATTGGACAGC |
|  | AACACTACCCAGGCATGATCCTCA |

**Table S6.** Sequences of shRNAs used in the study.

| **Clone ID** | **Target sequence** |
| --- | --- |
| ASN0000000003 | CCTAAGGTTAAGTCGCCCTCG |
| [TRCN0000239325](http://rnai.genmed.sinica.edu.tw/cloneInfo/cloneId/TRCN0000239325) | TAGCTTATCCTCGAACCTTAT |
| [TRCN0000239326](http://rnai.genmed.sinica.edu.tw/cloneInfo/cloneId/TRCN0000239326) | TCCACCCTCAGGCCTTATAAT |
| [TRCN0000239327](http://rnai.genmed.sinica.edu.tw/cloneInfo/cloneId/TRCN0000239327) | CTGGTCTGCAAACCTTACTAG |
| [TRCN0000239328](http://rnai.genmed.sinica.edu.tw/cloneInfo/cloneId/TRCN0000239328) | CTCGAACCTTATGAGACATAG |
| [TRCN0000257373](http://rnai.genmed.sinica.edu.tw/cloneInfo/cloneId/TRCN0000257373) | GCCTTTAGCTGGTCTGCAAAC |

**Table S7.** Primer sequences for ChIP assay in this study.

| **Gene name** | **Sequence** | **Site and Amplicon** |
| --- | --- | --- |
| *MT-1M* | CAAGGCGGGGAAGGAGGAGAAA | -343~-600 (167 bp) |
|  | TCGCCCCGCCGTTTTCAACT |  |
| *MT-1G* | GGGTGTAGCAGGCAACCTCA | -199~-358 (160 bp) |
|  | GGAGATGGGCCAAGTGCAAG |  |
| *MT-1H* | GAAGTGGCACTGCTTGGTGT | -846~-690 (157 bp) |
|  | TGTCTCTGTTTTGCTTCCAG |  |
| *GAPDH* | CGCTCTCTGCTCCTCCTGTT | 260 bp |
|  | CCATGGTGTCTGAGCGATGT |  |
| *MYT1* | GAGGATGGCTTTTTGAAACTGCTC | intron (170 bp) |
|  | TTGATATGCGCTCCCCTTCC |  |

**Fig. S1.** Huh-7 and HepG2 cells were transiently transfected with control vector and 14-3-3ε–overexpression vector (tagged with flag) for 48 h. Relative expression of MT-1 isoforms were determined by western blot (left panel, actin was used as loading control) and qPCR analysis (right panel, normalized to both control cells as well as *GAPDH*). Letters represent MT-1A, -1E, -1F, -1G, -1H, -1M and -1X, respectively. Scale bars: mean ± SD. *, *P* < 0.05; **, *P* < 0.01.

**Fig. S2.** Expression of MT-1 isoforms by transfection of 14-3-3ε siRNA. Scramble and 14-3-3ε siRNAs were transfected into 14-3-3ε-overexpressing cells for 48 h. Relative expression of 14-3-3ε and MT-1 isoforms were determined by qPCR (left panel, normalized to both control cells as well as *GAPDH*) and western blot analysis (right panel, actin was used as loading control). Scale bars: mean ± SD. **, *P* < 0.01.

**Fig. S3.** 14-3-3ε cells were transfected with or without peptide-based 14-3-3 inhibitor, difopein for 48 h. Relative expression of MT-1 isoforms were determined by western blot (left panel, actin was used as loading control) and qPCR analysis (right panel, normalized to both control cells as well as *GAPDH*). Letters represent MT-1A, -1E, -1F, -1G, -1H, -1M and -1X, respectively. Scale bars: mean ± SD. **, *P* < 0.01.

**Fig. S4.** 14-3-3ε cells were transiently transfected with MT-1M overexpression vector. Cell growth was examined by anchorage-independent analysis. Scale bars: mean ± SD. **, *P* < 0.01.

**Fig. S5**. Effect of pharmacological inhibitors on MT-1 isoform expression. 14-3-3ε–overexpressing stable cells were treated with pharmacological inhibitors (1 μM SB216762, 10 μM Y27632 and 30 μM SP600125) for 24 h. Expression of MT-1 isoforms was examined by qPCR (normalized to both control cells as well as *GAPDH*). Scale bars: mean ± SD. *, *P* < 0.05; **, *P* < 0.01.

**Fig S6.** **a** Relative expression of phosphorylated-ERK1/2, total ERK1/2, phosphorylated Akt, total Akt and β-catenin were examined by western blotting analysis in control and 14-3-3ε-overexpressing cells. Actin was used as loading control of western blotting analysis. **b,c** Huh-7 cells were treated with Wnt signaling inhibitor, DKK-1 for 24 h. Expression of MT-1 were determined analyzed by **b** western blotting (actin was used as loading control) and **c** qPCR (normalized to both control cells as well as *GAPDH*). Scale bars: mean ± SD. *, *P* < 0.05; **, *P* < 0.01.

**Fig. S7**. 14-3-3ε–overexpressing cells were treated with 10 μg/μl U0126, 10 μM rapamycin, 20 μM LY294002 or DMSO for 24 h. Colony formation analysis of 14-3-3ε–overexpressing cells after treatment with various concentrations of U0126, rapamycin, LY294002 or DMSO. Scale bars: mean ± SD. *, *P* < 0.05; **, *P* < 0.01.

**Fig. S8.** Relative expression of ZNF479, EGR1 and ZNF578 were determined by **a** qPCR and **b** western blot analysis in 14-3-3ε–overexpressing cells. Relative expression of ZNF479, EGR1 and MT-1 were determined by **c** qPCR (left panel) and **d** western blot analysis (right panel) in PDTC treated cells. **e,f** 14-3-3ε–overexpressing cells were transfected with EGR1 siRNA for 48 h. **e** Relative expression of EGR1 and MT-1 were determined by qPCR (left panel) and **f** western blot analysis (right panel). Scale bars: mean ± SD. *, *P* < 0.05. **, *P* < 0.01. Gene expression of qPCR analysis was normalized to both control cells as well as *GAPDH*. Actin was used as loading control for western blotting analysis.

**Fig. S9**. shRNA-reduced expression of ZNF479 in Huh-7 cells. Scramble and ZNF479 shRNAs were transfected into Huh-7 cells, and stable cells were selected by 2 μg/ml puromycin for 4 weeks. Knockdown efficiency of different clones examined by western blot analysis. Actin was used as loading control.

**Fig. S10.** Expression of ZNF479, MT-1, ASH2L, Menin, and DNMT1 in PDTC-treated tumors. 14-3-3ε-overexpressing cells were subcutaneously injected into 8-week-old BALB/c nu/nu nude mice. At 1 week after injection, vehicle control (PBS) or PDTC (50 and 100 mg/kg) was peri-tumorally injected into mice every 2 days for 4 weeks. PBS or PDTC-treated tumors from mice were homogenized and expression was examined by **a** western blot analysis and **b** quantified with normalization to actin level. *, *P*<0.05; **, *P* < 0.01.

**Fig. S11.** Expression of UHRF1, DNMT1, ASH2L, Menin, ZNF479, MT-1 and GAPDH determined by western blotting analysis in HCC patients with HBV. T: tumor; N: non-cancerous/normal tissue. GAPDH was used as loading control.

**Fig. S12.** Relative expression of ZNF479, MT-1, DNMT1, UHRF1, ASH2L and Menin were determined by western blotting analysis in HCC patients with HBV. Relative protein expression was normalized with GAPDH.

**Fig. S13.** The binding efficacy of H3K4me3 on the *MT-1M, MT-1G* and *MT-1H* promoters were performed by ChIP analysis in control and ZNF479-overexpressing cells using specific antibodies against H3K4me3 or IgG control. Immunoprecipitated DNA was amplified by **a** PCR and **b** qPCR with primers for *MT-1M, MT-1G* and *MT-1H* promoters (Table S7). *MYT1* and *GAPDH* represented as control in this experiment. Scale bars: mean ± SD. *, *P* < 0.05; **, *P* < 0.01.

**Fig. S14.** Expression of H3R2me2 in 14-3-3ε– and ZNF479–overexpressing cells. Protein level of H3R2me2 was determined in **a** control/14-3-3ε–overexpressing stable cells; **b** Huh-7 cells transiently transfected with ZNF479-overexpression plasmid and **c** ZNF479 siRNA for 48 h. Expression of H3R2me2 determined by western blot analysis. Actin was used as loading control.

**Fig. S15.** Expression of ZNF479 in MT-1 overexpressing cells. Huh-7 cells were transiently transfected with MT-1M, MT-1G, MT-1H and control vectors for 48 h. Expression of ZNF479 was determined by western blotting analysis. Actin was used as loading control.

**Fig. S16.** HepG2 cells were transfected with the scramble and ZNF479 siRNAs for 48 h. Expression of ZNF479, MT-1, H3K4me2, H3K4me3, ASH2L, Menin, DNMT1 and UHRF1 determined by western blot analysis. Actin was used as loading control.

**References**

1 Xu, Z. *et al.* A role of histone H3 lysine 4 methyltransferase components in endosomal trafficking. *The Journal of cell biology* **186**, 343-353, doi:10.1083/jcb.200902146 (2009).

2 El-Serag, H. B. & Rudolph, K. L. Hepatocellular carcinoma: epidemiology and molecular carcinogenesis. *Gastroenterology* **132**, 2557-2576, doi:10.1053/j.gastro.2007.04.061 (2007).

3 El-Serag, H. B., Marrero, J. A., Rudolph, L. & Reddy, K. R. Diagnosis and treatment of hepatocellular carcinoma. *Gastroenterology* **134**, 1752-1763, doi:10.1053/j.gastro.2008.02.090 (2008).

4 Bruix, J. *et al.* Clinical management of hepatocellular carcinoma. Conclusions of the Barcelona-2000 EASL conference. European Association for the Study of the Liver. *J Hepatol* **35**, 421-430 (2001).

5 Xu, B. *et al.* Menin promotes hepatocellular carcinogenesis and epigenetically up-regulates Yap1 transcription. *Proc Natl Acad Sci U S A* **110**, 17480-17485 (2013).
